# Adaptive Grouping Cloud Model Shuffled Frog Leaping Algorithm for Solving Continuous Optimization Problems

**DOI:** 10.1155/2016/5675349

**Published:** 2015-12-27

**Authors:** Haorui Liu, Fengyan Yi, Heli Yang

**Affiliations:** ^1^School of Automotive Engineering, Dezhou University, Dezhou 253023, China; ^2^Automotive Engineering College, Shandong Jiaotong University, Jinan 250023, China

## Abstract

The shuffled frog leaping algorithm (SFLA) easily falls into local optimum when it solves multioptimum function optimization problem, which impacts the accuracy and convergence speed. Therefore this paper presents grouped SFLA for solving continuous optimization problems combined with the excellent characteristics of cloud model transformation between qualitative and quantitative research. The algorithm divides the definition domain into several groups and gives each group a set of frogs. Frogs of each region search in their memeplex, and in the search process the algorithm uses the “elite strategy” to update the location information of existing elite frogs through cloud model algorithm. This method narrows the searching space and it can effectively improve the situation of a local optimum; thus convergence speed and accuracy can be significantly improved. The results of computer simulation confirm this conclusion.

## 1. Introduction

Shuffled frog leaping algorithm (SFLA) proposed by Eusuff and Lansey [1] has the advantages of being easy to implement and having fast speed and global optimization capability and has been widely used in many areas. Elbeltagi et al. [2] proved that SFLA is superior to genetic algorithms which approximates particle swarm algorithm in success rate and convergence rate when solving some continuous optimization problems. Amiri et al. [3] improved *K*-means clustering method by using SFLA and the result showed that the new method is superior to other clustering methods such as ant colony optimization (ACO) in solution quality and speed. However, SFLA has shortcomings of premature, slow convergence and less precision, so that it is not ideal in solving high-dimensional continuous optimization problem. The main cause of the defect is that, in the later stage of evolution, population diversity declines rapidly, and the method lacks locally refined search capabilities. In order to improve the performance of SFLA optimization algorithms, many scholars [4–36] improved parameter adjustment and memeplexes update mode and combined SFLA with other intelligent algorithms, and so forth, and achieved good optimization results.

The cloud mode [37–40] is proposed to represent the transformation of the uncertainty between the qualitative concept and quantitative value and describes the qualitative knowledge. It is also being widely used in the fields such as the fuzzy evaluation and intelligent control. Characterized by the coexistence of the uncertainty and certainty as well as the stability during the course of knowledge representation, the cloud model has reflected the fundamental principle of the biological evolution in nature.

Some groups of animals in the foraging process have two roles: either looking for prey or sharing current best prey. “Elite frog” finds current best prey, and “variation frog” is involved in foraging and shares “elite frog” results. “Elite frog” may be fooled by illusion which corresponds to local optimal solution in intelligent optimization problems. Variation frog is in charge of discovering prey in the vicinity or a new search track and helps “elite frog” to avoid being confused by some illusion and leaving foraging groups into local optimum. “Elite frog” and “variation frog” guide groups to better search trajectory and find their prey eventually.

In summary, the existing research has made many achievements. However, convergence speed and optimization accuracy need to be improved; particularly when solving multimode function the SFLA easily falls into local optima. This paper presents a continuous optimization algorithm based on grouping SFLA optimization; the goal is to improve the convergence speed and accuracy of the optimization process, especially to improve the optimizing performance for multimode function. Firstly, the cloud model is used to update the existing elite frog. Random uniform design method (RUDM) is used to construct a frog of variation group. Adaptive grouping algorithm is used to determine the content of each group. Optimization is completed through several iterations. A large number of simulation experiments confirm that the algorithm has high speed and optimization precision.

## 2. Basic Definitions and Proposed Algorithm

### 2.1. Basic SFLA

Shuffled frog leaping algorithm (SFLA) is a swarm evolution algorithm which imitates frogs exchanging information as the divided memeplexes when they are searching for food. The combination of global search and local search in memeplex develops the algorithm to approach the optimal solution [26].

In SFLA, memeplex is virtually composed of frogs which represent a candidate solution. The population is divided into several memeplexes each of which includes a certain amount of frogs. Different memeplexes have their own culture as well as the behavior which can be influenced by each other. All memeplexes will be mixed up to form a new population after the local search has been implemented for defined times for each of them, which can exchange information globally among all the memeplexes [3, 28]. Alternate the global and local search until the information desired convergence is reached which is expressed as certain convergence accuracy or the maximum of iterations. Solution steps of unconstrained function optimization are as follows.

*(1) Initialization*. *p* candidate solution can be generated in its feasible domain *Ω* ⊂ **R** ^*D*^ for a *D*-dimensional problem. The *p* candidate solution can be expressed as initial swarm *S* = (*X*_1_, *X*_2_,…, *X*_*p*_) in which *X*_*i*_ = (*x*_*i*1_, *x*_*i*2_,…, *x*_*iD*_) descries the *i*th (1 ≤ *i* ≤ *p*) candidate solution. 

*(2) Memeplex Classification*. Partition the population into *m* memeplexes as follows: allocate the frogs to the groups according to the fitness values. The first frog with the highest value moves to the first memeplex, the second highest frog moves to the second memeplex, and the *m*th highest frog moves to the last memeplex. Then, *m* + 1th frog moves to the first memeplex again. These operations continue until the last frog is allocated to a memeplex. Finally, each memeplex contains *n* frogs. Thus, *p* = *n* × *m*. 

*(3) Local Search in Memeplex*. Let the best frog of a memeplex be *X*_*b*_, let the worst one be *X*_*w*_, and let the global best one be *X*_*g*_. The search in each memeplex is to renew *X*_*w*_ of the memeplex in the following strategy:

(1) Di=min⁡intrXb−Xw,Dmax,rXb−Xw≥0max⁡intrXb−Xw,−Dmax,rXb−Xw≤0

(2) Xneww=Xw+Di.

In the function above, *D*_*i*_ is renewing value of step size; int[*x*] is roundness of *x*; *r* is the random number in the range of (0,1) whose effective bit depends on problems and simulation environment; *D*_max_ is the maximum distance allowing frog moving.

If there exists *X*_new*w*_ ⊂ *Ω*, after update, then substitute *X*_*w*_ with *X*_new*w*_. Otherwise replace *X*_*b*_ in Function (1) with *X*_*g*_. The new *X*_new*w*_′ is computed by Functions (1) and (2). If *X*_new*w*_′ ⊂ *Ω* and *F*(*X*_new*w*_′) < *F*(*X*_*w*_) exist, then substitute *X*_*w*_ with *X*_new*w*_′; otherwise a new arbitrary candidate solution is generated to replace *X*_*w*_. The iteration will not end until the search of the designed search time has been reached. 

*(4) Global Information Exchange*. After the local search is completed, all the memeplexes are mixed into one swarm which is then operated as the methods shown in steps (2) and (3) until the result meets the termination criterion.

The termination criteria for the algorithm could be satisfied by one of the following three conditions:

(1) The value of the main objective function *F* reaches an acceptable and optimum value.
(2) The number of iterations reaches a predefined value (*N*); it varies for different number of dimensions in a problem.
(3) During several consecutive iterations, no progress could be seen in the value of the main objective function *F*.

**Figure 1:**
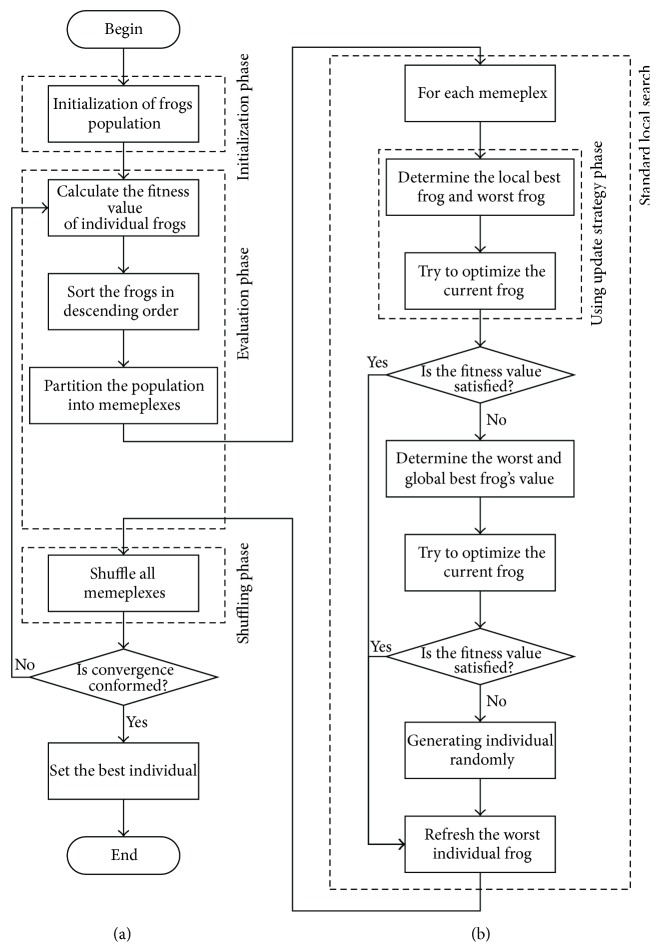
(a) The standard SFLA algorithm's main diagram; (b) local search for each memeplex.

The standard SFLA's diagram is shown in Figure 1.

### 2.2. Cloud Model

Cloud model provides a solution for the problem in combination with both qualitative and quantitative concept, which realizes the conversion between qualitative concept and quantitative concept and shows the fuzziness and randomness of concept expressing. Let *U* be a universe in accurate value, which corresponds to a qualitative concept *A* and could be one-, two-, or several-dimensional. There exists a random value satisfying the formula of *y* = *U*_*A*_(*x*) steadily for an arbitrary element in the universe, reflecting the certainty degree of *x* corresponding to the concept *A*. The distribution of *x* in the universe of *U* is called cloud model or cloud for short.* x* is denoted as cloud droplet in universe space [37].

**Figure 2:**
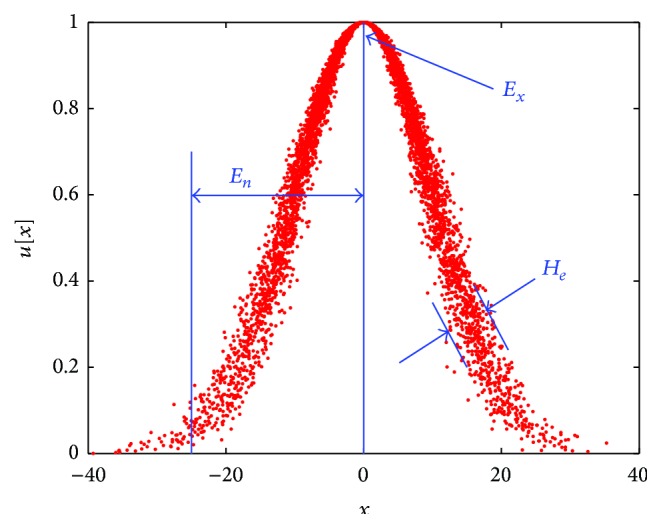
Digital features of cloud model.

Expectation *E*_*x*_, entropy *E*_*n*_, and hyperentropy *H*_*e*_ describe the digital characteristics of cloud, and they are illustrated in Figure 2. Expectation *E*_*x*_ is the most typical sample point relating to concept *A*. Entropy *E*_*n*_ represents the uncertainty of the concept *A*, revealing both the acceptance range of universe for languages and the randomness of forming cloud droplet. The higher *E*_*n*_ value expresses the wider range of cloud droplet generating and the stronger randomness. Hyperentropy *H*_*e*_ is measurement of *E*_*n*_. In other words, *H*_*e*_ is the entropy of *E*_*n*_.

The algorithm of the basic normal cloud generator is operated in following steps.

Firstly, a random number *E*_*n*_ is achieved taking *E*_*n*_ as expectation and *H*_*e*_ as standard deviation.

Secondly, a random number *x* is achieved taking *E*_*x*_ as expectation and absolute value of *E*_*n*_ as standard deviation. *x* is the cloud droplet.

Thirdly, *y* is calculated in the formula as follows:

(3) $$\begin{array}{c} y=e^{{-\left(x-Ex\right)}^{2}/2{\left(En^{\prime }\right)}^{2}} \end{array}$$

which is certainty degree of qualitative concept *A*.

Finally, the first, second, and third step are repeated until cloud droplet number reaches object value.

3*E*_*n*_ rule of basic normal cloud can be described as follows. Among all the cloud droplets that make contribution to qualitative concept *A*, 99.7% of them are located in the range of [*E*_*x*_–3*E*_*n*_, *E*_*x*_+3*E*_*n*_]; thus cloud droplets outside this range only have a tiny possibility of making contribution to qualitative concept *A* which can be ignored [38–40].

### 2.3. Adaptive Grouping Cloud Model SFLA

An important reason of the existing algorithms easily falling into local optimum is as follows: when the optimization process reaches a local bottom or peak, the algorithm lacks mechanisms to jump out of the local optima.

**Figure 3:**
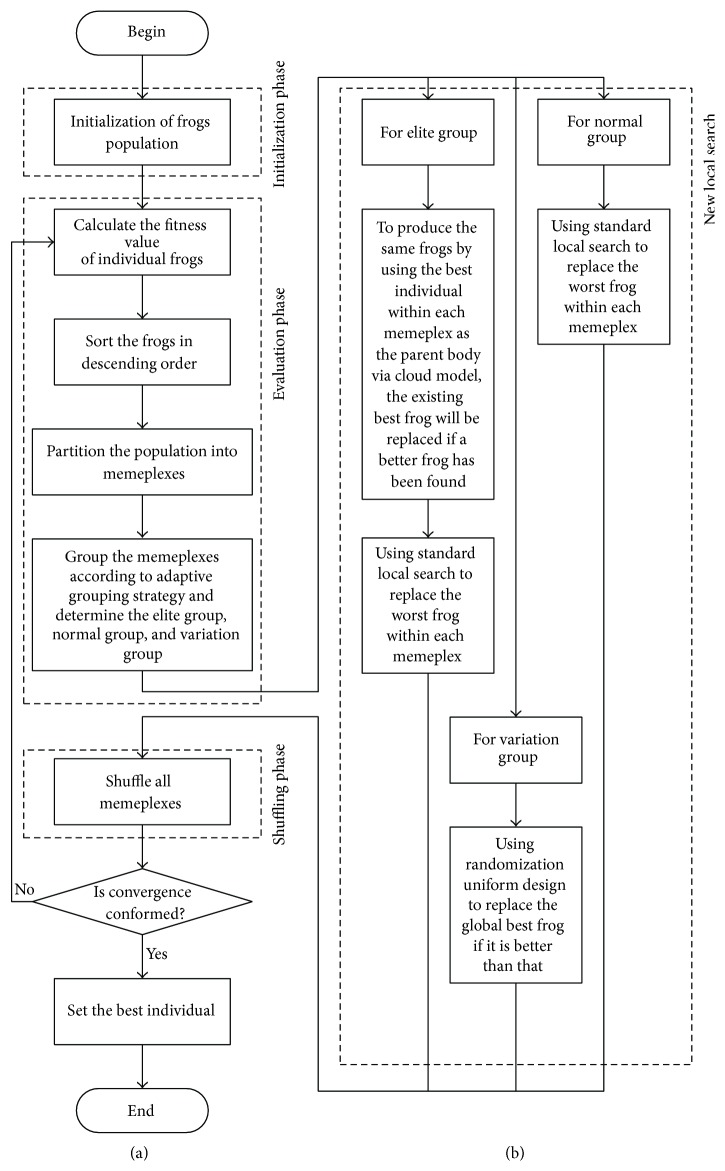
(a) The proposed SFLA algorithm's main diagram; (b) local search for each memeplex.

The grouping optimization algorithm makes the other groups work regularly when one group runs into local optimum and that greatly reduces search space so that the optimization process avoids local optima and laid the foundation for the parallel algorithms. The grouping optimization algorithm divides definition region into three groups and then assigns certain memeplexes to each group. The adaptive grouping cloud model SFLA's diagram is shown in Figure 3.

#### 2.3.1. Relevant Definition

*Group* includes some memeplexes.

*Elite group* has bigger fitness and its memeplexes may have more excellent next generation by using cloud model.

*Normal group* uses standard local search.

*Variation group* guarantees any possibility of evolution, and only a few individuals of that will undergo mutation during evolution.

#### 2.3.2. Elite Group Policy

In the search process, the “elite policy” means that the best individual in each generation is defined as the elite frog and kept to the next cloud model generation and used to update the location best frog and accelerate the convergence rate. When each memeplex is updated, the worst individual will be updated, as well as the best individual in memeplex. The worst individual in memeplex will be updated by traditional frog leaping algorithm and the best individual by the normal cloud model algorithm in Section 2.2. The best individual in memeplex will be taken as a normal cloud droplet, and it is used to produce a group of cloud droplets with the same number as the memeplex, that is, a group of frogs. In this new memeplex, if a frog is better than the best individual within the original memeplex, the new best individual will replace the old one; otherwise it remains the same. Specific generation algorithm is as follows: *E*_*x*_ is the center of new individuals generation; the best individual in memeplex will be regarded as *E*_*x*_. Take modern fitness variance *σ* ^2^ as *E*_*n*_, dynamical changing search scope; take *E*_*n*_/*T* as *H*_*e*_ [41], and *T* can be selected from 3 to 6.

#### 2.3.3. Variation Group Policy

Using random uniform design method [42] construct the initial population. Random uniform design (RUD) is using generally generated vector replacing primitive roots of uniform design generating randomized uniform design points set. The deviation of many randomized uniform design points set is obviously smaller than the original uniform design; thus it has better representativeness. Therefore, in this paper more uniform variant frogs constructed by randomized uniform design method are adopted, replacing the existing best frog.

#### 2.3.4. Adaptive Grouping Policy

Frogs with better adaptive fitness are divided into the elite group, and those with poor adaptive fitness are always divided into variant group. The adaptive fitness grouping method is given as follows.

**Step 1 .**

Figure out adaptive value's average fitness *N*_avg_ of the best individuals in all memeplexes.

**Step 2 .**

Figure out adaptive value's average fitness *N*_l-avg_ of the best individuals whose adaptive value is larger than *N*_avg_ in all memeplexes.

**Step 3 .**

Figure out adaptive value's average fitness *N*_s-avg_ of the best individuals whose adaptive value is smaller than *N*_avg_ in all memeplexes.

**Step 4 .**

Figure out the number of memeplexes whose best individuals' adaptive fitness is larger than *N*_l-avg_, *N*_elite_, constructing the elite group.

**Step 5 .**

Figure out the number of memeplexes whose best individuals' adaptive fitness is smaller than *N*_s-avg_, *N*_variation_, constructing the variant group.

**Step 6 .**

The rest belong to normal group.

## 3. Results and Discussion 

By taking three-function extreme optimization as example, a contrast is conducted through the particle swarm optimization (PSO), quantum ant colony algorithm (QACA), grouping ant colony algorithm (GACA), cloud model shuffled frog leap algorithm (CM-SFLA), and adaptive grouping chaos cloud model frog leap algorithm (AGCM-SFLA), so as to verify the optimum performance of the algorithm. The parameter setting of frog leap algorithm is as follows: the population size is 300. It is divided into 15 memeplexes, each memeplex having 20 frogs. The iteration time inside each memeplex is 15, and the maximum iterative algebra is 6000. Each function runs for 30 times independently. The error precision is 10^−10^. Three functions are listed in Table 1 to compare the optimal result, worst result, average result, average time, and variance of the five algorithms: *f*_1_ is a two-dimensional complex function with numerous minimum points. The minimum value is 0. *f*_2_ has six local optimal solutions and two global minimum points. *f*_3_ is a multipeak function. Its global minimum value is 0. The operational simulation result comparison of function* f*_1_~*f*_3_ is shown in Tables 2, 3, and 4.

As can be seen from the simulation results, the adaptive grouping cloud model shuffled frog leap algorithm (AGCM-SFLA) proposed by this paper can achieve good solution precision and solution speed. There are two main reasons below for that.

(1) Seen from the best result, the worst result, and the average result, the optimization search effect of PSO is the worst, followed by QACA, GACA, and CM-SFLA. AGCM-SFLA is the best. Although the calculation results of *f*_2_ by reserving four decimal points are the same, AGCM-SFLA is closer to the global optimum value from the perspective of the actual calculation precision. This is because the species initialization of the reserve study mechanism is adopted by the algorithm, which increases the opportunity for individuals to get close to the optimum solution. Moreover, the stable tendency of the cloud model can well protect the optimum individual, thus realizing the adaptive positioning of the surrounding optimum value. The individual diversity can be maintained, while the evolution speed of algorithm acceleration and the search of optimum efficiency can be achieved by randomness. Meanwhile, the introduced random uniform design (RUD) can help the algorithm to avoid local optimum in the later period, which is conductive to achieving the global optimum solution and preventing defects such as earlier restraint, slow restraint, and regional optimum of some complex functions.

(2) Seen from the average restraint and iterative algebra, the running time, and the variance, SFLA iteration time is less than that of PSO. If the iteration algebra is considered inside the frog leap algorithm memeplex, the iteration algebra of SFLA is more. However, seen from the running time, SFLA is faster. This is because only the worst particles are renewed in iteration by SFLA. Under the best circumstance, once iteration is only calculated once, the worst situation is only calculated three times. Relative to the particle groups, the calculation numbers of all particles in each time of iteration renewal are fewer, so the speed can be fast. Furthermore, the operation time and variance of AGCM-SFLA are both superior to the previous algorithms. The calculation result of function *f*_3_ is optimal, indicating the AGCM-SFLA has strong adaptability for the solution of high-dimensional multipeak functions.

## 4. Conclusions 

By using the cloud model algorithm, this paper refines the restraint area of the “elite frog” group to discover the global optimum position; the RUD is used to overcome the restrictive advantages caused by the irrational position distribution in the intelligent algorithm. Global optimum search is conducted for the “ordinary frogs” space along with the avoidance of the global optimum position. In this way, the optimum solution restraint can be done quickly, thus avoiding problems such as the local optimum solution and early restraint caused by classic frog leap algorithm. According to the simulation result, the proposed algorithm is with advantages such as high precision and fast restraint speed. The effective integration of the cloud model and the evolution calculation can help to expand the application field of the cloud model, which also promotes the new exploration and trial for the research about the evolution calculation.

## Figures and Tables

**Table 1:** Tested function.

Number	Function	Interval of *x*_*i*_
*f*_1_	$f_{1}=0.5+\frac{\sin^{2}\left(\sqrt{x_{1}^{2}+x_{2}^{2}}\right)-0.5}{{\left[1+0.001\left(x_{1}^{2}+x_{2}^{2}\right)\right]}^{2}}$	−100 ≤ *x*_*i*_ ≤ 100

*f*_2_	$f_{2}=\left[4-2.1x_{1}^{2}+\frac{1}{3}x_{1}^{4}\right]x_{1}^{2}+x_{1}x_{2}+\left(-4+4x_{2}^{2}\right)x_{2}^{2}$	−3 ≤ *x*_1_ ≤ 3, −2 ≤ *x*_2_ ≤ 2

*f*_3_	$f_{i}=10\times n+\overset{n}{\underset{i=1}{\sum }}\left[x_{i}^{2}-10\cos\left(2\pi x_{i}\right)\right]$	−5 ≤ *x*_*i*_ ≤ 5, *n* = 10

**Table 2:** Simulation results of function *f*_1_.

Algorithm	Optimal results	Worst result	Result	Time/s	Variance
PSO	1.55166*e* − 12	0.01067	10.6975*e* − 04	0.286	7.01*e* − 06
QACA	8.93574*e* − 12	10.27554*e* − 11	6.3734*e* − 11	0.199	4.66*e* − 21
GACA	6.36979*e* − 12	10.73633*e* − 11	4.85551*e* − 11	0.187	4.12*e* − 21
CM-SFLA	4.53099*e* − 12	7.89415*e* − 11	3.9303*e* − 11	0.179	2.64*e* − 21
AGCM-SFLA	5.03124*e* − 13	8.9265*e* − 11	3.04139*e* − 11	0.165	2.11*e* − 21

**Table 3:** Simulation results of function *f*_2_.

Algorithm	Optimal results	Worst result	Result	Time/s	Variance
PSO	−1.135745	−1.135745	−1.135745	1.699	2.35*e* − 19
QACA	−1.137994	−1.137994	−1.137994	1.728	4.11*e* − 21
GACA	−1.1391185	−1.1391185	−1.1391185	1.646	3.95*e* − 21
CM-SFLA	−1.140243	−1.140243	−1.140243	1.011	3.98*e* − 21
AGCM-SFLA	−1.1413675	−1.1413675	−1.1413675	0.925	2.31*e* − 21

**Table 4:** Simulation results of function *f*_3_.

Algorithm	Optimal results	Worst result	Result	Time/s	Variance
PSO	6.56667	44.87252	19.48122	13.244	3.90*e* + 02
QACA	5.24447*e* − 11	2.18889	0.65692	4.522	9.89*e* − 01
GACA	7.49716*e* − 11	5.66808*e* − 10	9.00064*e* − 10	3.671	6.91*e* − 01
CM-SFLA	2.66002*e* − 11	10.78605*e* − 11	7.58483*e* − 11	2.191	4.54*e* − 21
AGCM-SFLA	10.31712*e* − 12	9.76371*e* − 11	5.70064*e* − 11	1.216	3.89*e* − 21
